# A comprehensive dose evaluation project concerning animals affected by the Fukushima Daiichi Nuclear Power Plant accident: its set-up and progress

**DOI:** 10.1093/jrr/rrv069

**Published:** 2015-12-18

**Authors:** Shintaro Takahashi, Kazuya Inoue, Masatoshi Suzuki, Yusuke Urushihara, Yoshikazu Kuwahara, Gohei Hayashi, Soichiro Shiga, Motoi Fukumoto, Yasushi Kino, Tsutomu Sekine, Yasuyuki Abe, Tomokazu Fukuda, Emiko Isogai, Hideaki Yamashiro, Manabu Fukumoto

**Affiliations:** 1 Department of Pathology, Institute of Development, Aging and Cancer, Tohoku University; 2 Graduate School of Science, Tohoku University; 3 Institute for Excellence in Higher Education, Tohoku University; 4 National Research Center for Protozoan Diseases, Obihiro University of Agriculture and Veterinary Medicine; 5 Graduate School of Agricultural Sciences, Tohoku University; 6 Faculty of Agriculture, Niigata University

**Keywords:** Fukushima Daiichi Nuclear Power Plant, sample bank, archives, radiation effects, animals

## Abstract

It is not an exaggeration to say that, without nuclear accidents or the analysis of radiation therapy, there is no way in which we are able to quantify radiation effects on humans. Therefore, the livestock abandoned in the ex-evacuation zone and euthanized due to the Fukushima Daiichi Nuclear Power Plant (FNPP) accident are extremely valuable for analyzing the environmental pollution, its biodistribution, the metabolism of radionuclides, dose evaluation and the influence of internal exposure. We, therefore, sought to establish an archive system and to open it to researchers for increasing our understanding of radiation biology and improving protection against radiation. The sample bank of animals affected by the FNPP accident consists of frozen tissue samples, formalin-fixed paraffin-embedded specimens, dose of radionuclides deposited, etc., with individual sampling data.

## INTRODUCTION

### Establishment of a sample bank of animals affected by the FNPP accident

Explosions at the Fukushima Daiichi Nuclear Power Plant (FNPP) occurred after the Great East Japan disaster, and they released a huge amount of artificial radioactive substances into the environment; most of them were dispersed on 15 March 2011. Since then, people globally have been concerned about the late effects of radiation decades after the accident; even the acute effects were not detected. The evacuation zone was defined on 22 April 2011 as within a 20-km radius of the FNPP, and since then no one has been allowed to enter the area without permission from the Anti-Disaster Headquarters. Since April 2012, rearrangements of the restricted areas, including the evacuation zone, have been performed several times. We, therefore, term the area within a 20-km radius of the FNPP the ex-evacuation zone. On 12 May 2011, the Prime Minister ordered the Governor of Fukushima to euthanize livestock within the evacuation zone, preventing people from eating meats contaminated with radionuclides. With the intention of increasing understanding of the biological and human effects of both internal and external radiation exposure and of the spatio-temporal changes occurring after radioactive contamination, the aim of this project was to establish an archive system, to accurately evaluate radiation doses to individual organs from euthanized animals and to the environment at the contaminated site, and to preserve sample materials with data for future generations. There is no doubt that the technology associated with radiation measurement and analysis of biological effects will develop further in the future. This archive will become crucial to our understanding of the effects of radiation on humans and on the earth.

## MATERIALS AND METHODS

### Sampling methods and establishment of a tissue bank

**Fig. 1.**
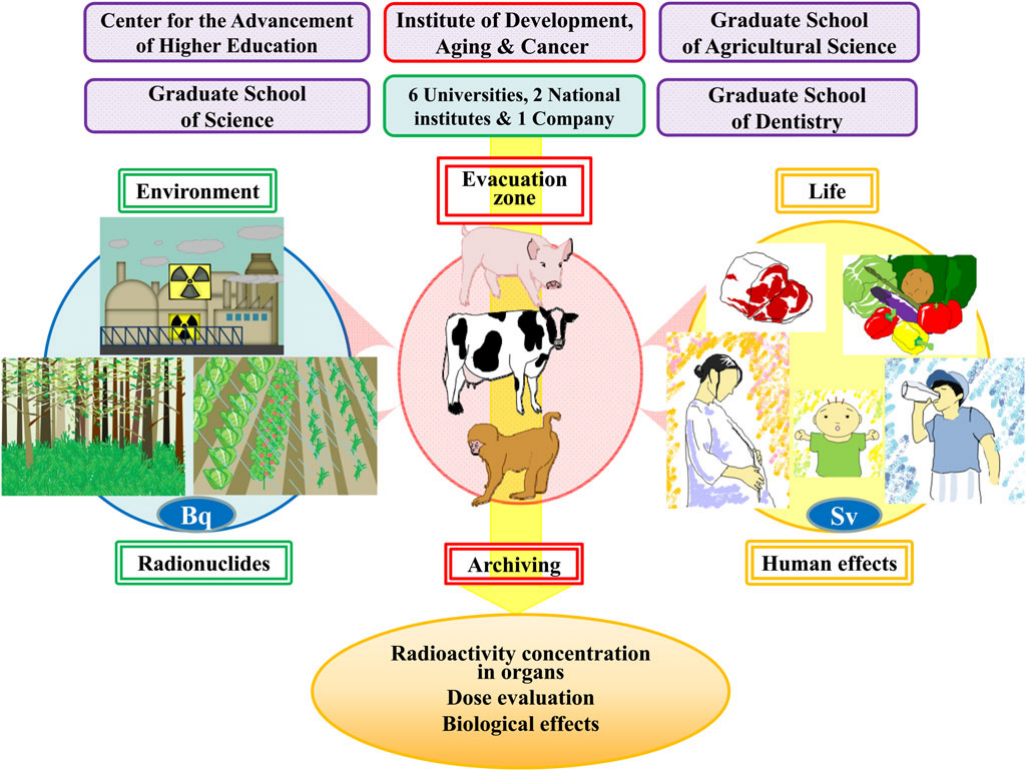
Comprehensive dose evaluation of animals within and around the ex-evacuation zone of the FNPP accident. This project cannot be accomplished without the development of science, the understanding of people, the opening of information, and adequate funding. A strong research group entitled the ‘Group for Comprehensive Dose Evaluation in Animals from the Area Affected by the FNPP Accident’ (composed of five faculties of Tohoku University, six other universities, two national institutes and a company specialized in chromosome analysis) has been established.

**Fig. 2.**
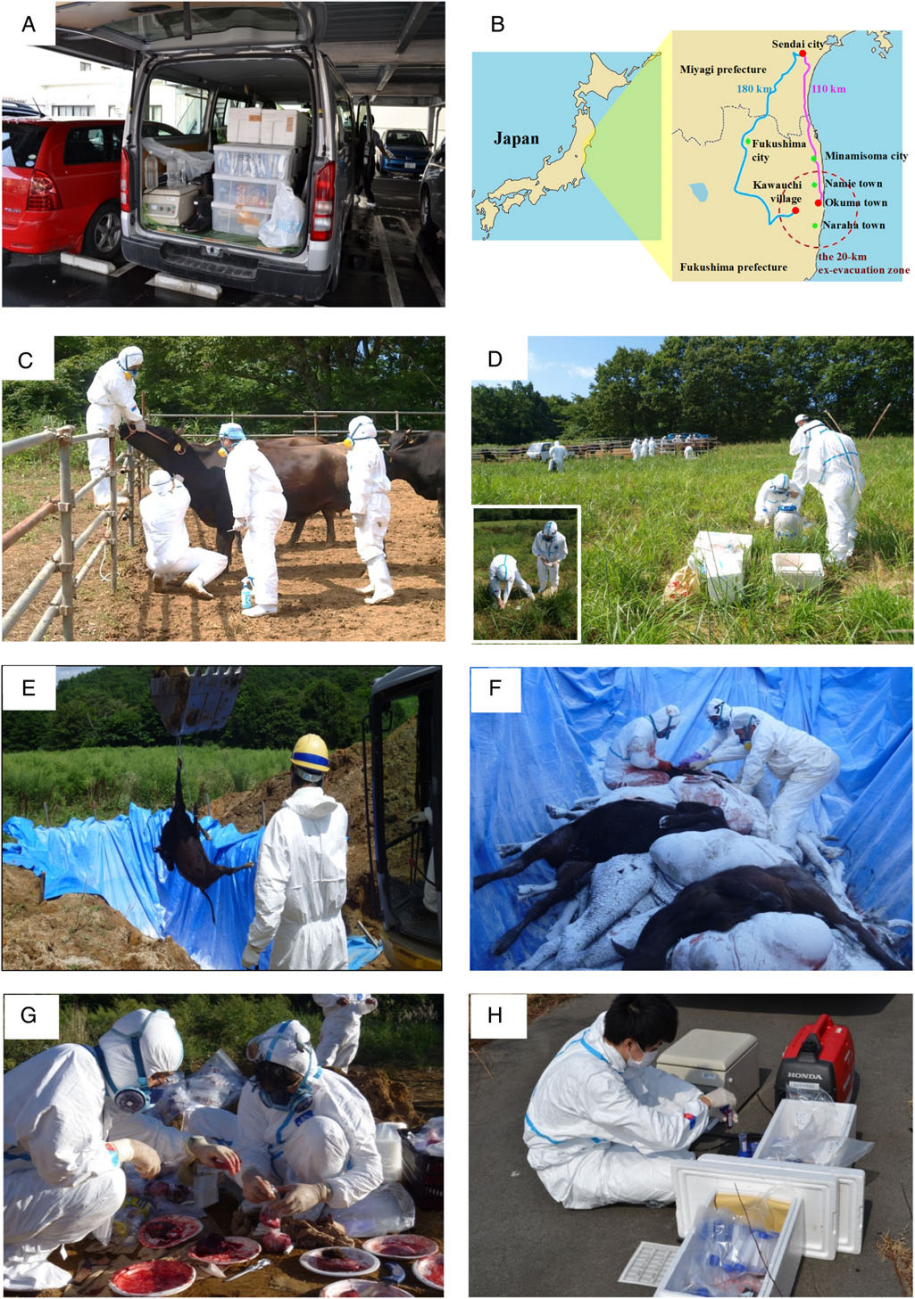
Sampling activity. A rent-a-car of van has been maintained since the beginning of this project and equipped with devices for dissection and sampling, including a portable freezer, a centrifuge, electric saws and a power generator (**A**). Sampling of cattle was started at Kawauchi village (where informed consent from farmers was first obtained) and ended at Okuma town. The roads in the mountainous area were disrupted by the earthquake, so the route taken was dependent on the sampling location (**B**). The unleashed cattle were trapped by food and water. After each animal was identified from its ear tag, it was anesthetized deeply and euthanized, using a muscle relaxant. Peripheral blood was taken from the jugular vein (**C**). While the veterinarians were performing the euthanasia, we collected grass with bate, and soil (**D**). The animal carcasses were brought to a ditch dug on public land (**E**), where we performed dissection (**F**). At this location, the organs were separated into four groups; one group destined for radioactivity measurement, two for cryopreservation in the various laboratories, and one for paraffin-embedded blocks (**G**). Cesium is believed to share transport pathways with potassium (such as the energy-dependent pump and channel). We therefore separated the blood cells from the plasma to avoid diffusion from one to the other during the time between sampling and radioactivity measurement (**H**).

**Fig. 3.**
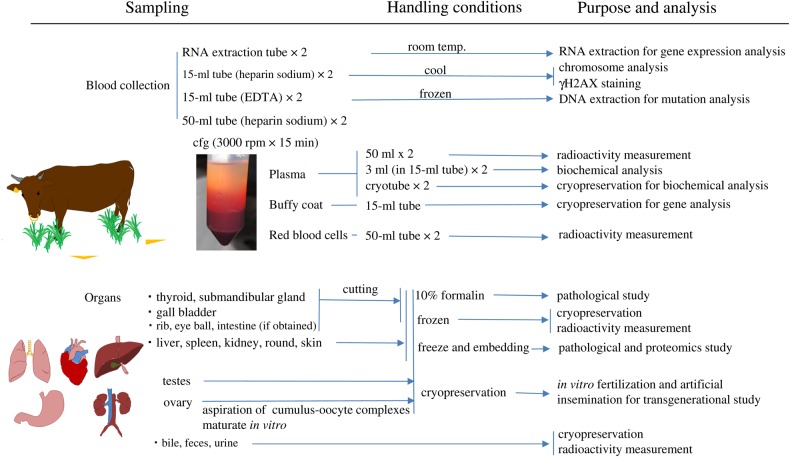
Handling and analysis of materials. Materials were divided up according to their intended analytical purpose.

We organized a research group entitled the ‘Group for Comprehensive Dose Evaluation in Animals from the Area Affected by the FNPP Accident’ and started the research activity after overcoming many difficulties and obstacles. We had to obtain finances for the project and also obtain permission to enter the ex-evacuation zone and to bring out organ samples (Fig. 1). Since 29 August 2011, we have been performing sampling of organs and peripheral blood from animals left behind in and around the ex-evacuation zone of the FNPP accident. Euthanasia and sampling began from the livestock about which consent was obtained from dairy farmers. After euthanasia was performed (by veterinarians from the Fukushima prefecture), peripheral blood was obtained from the jugular vein. Carcasses were carried by dump trucks to the burying place and put into the trench dug for burying them. Dissection was carried out in the trench, and the organs were divided into four groups: one for the measurement of radioactivity, one for histological examination and two for molecular study (Fig. 2). For molecular analysis, duplicates of frozen tissues were stored in two different laboratories (where electricity was supplied by two independent power companies) in case of power failure. We also separated the plasma from the blood cells on the dissection site. All samples were collected and divided according to the purpose of analysis (Fig. 3).

### Sample control via a barcode system

**Fig. 4.**
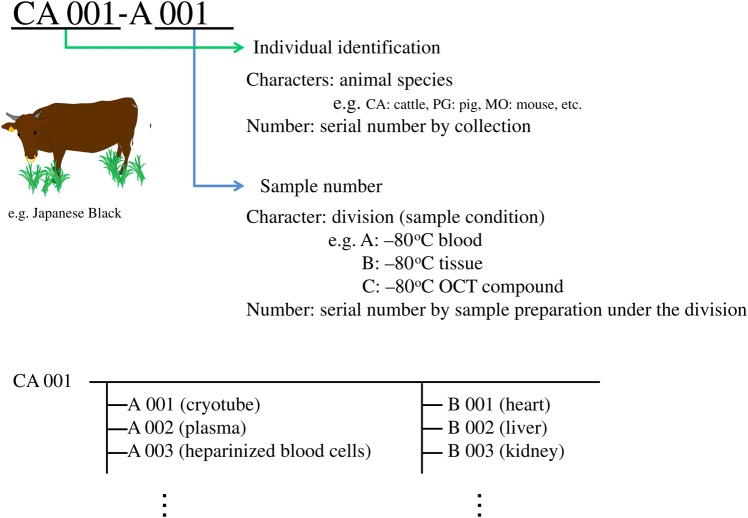
Sample control. All samples were itemized and issued with individual barcodes.

**Fig. 5.**
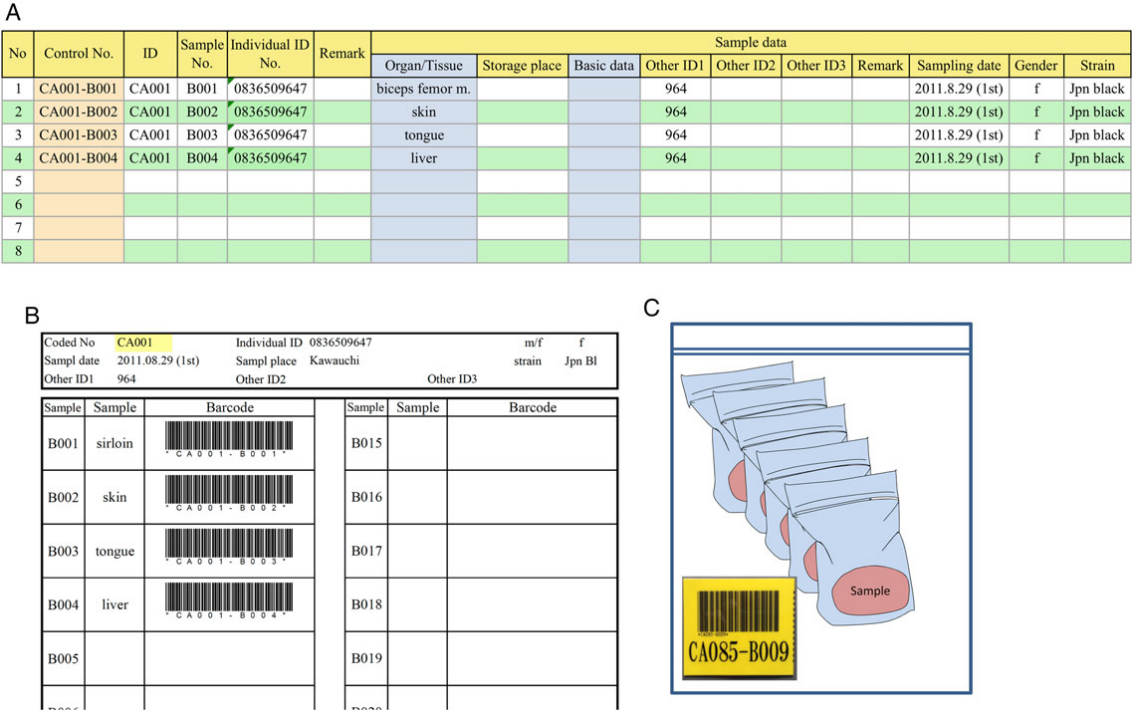
Connecting each sample and datum. A simple database was created using Excel (**A**). An information searching system was set up in conjunction with barcodes (**B**). Frozen tissue samples from each animal were stored in a single large freezer bag, which was labeled with the laminated barcode issued for that animal; tissue samples from the various organs were individually stored in small bags within the large bag (**C**).

All the materials were itemized and linked to their information via a barcode issued for each individual animal (Fig. 4). Each barcode was printed on a piece of tape by a PT-9800PCN desktop network thermal label printer (Brother Industries, Nagoya, Japan), using its attached software, P-touch editor. The barcode label was laminated and was put into a plastic freezer bag with the registered organ tissue samples associated with the particular animal. All sample data were compiled and organized into a chart on a Microsoft Excel spreadsheet controlled by a barcode system (Fig. 5).

## RESULTS AND DISCUSSION

**Fig. 6.**
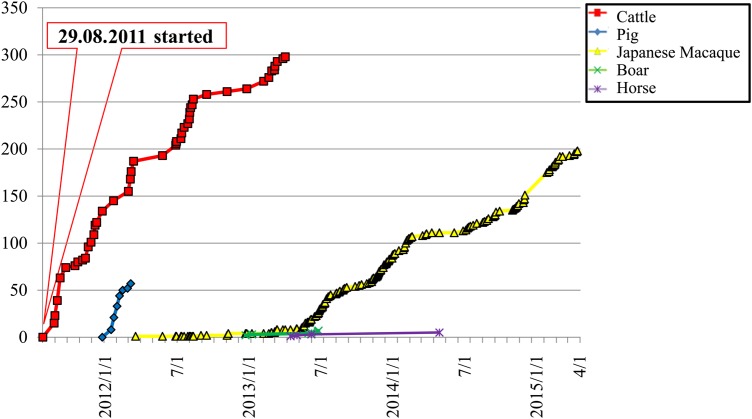
The cumulative number of samples. The project commenced on 29 August 2011 and is continuing. In total, we obtained samples from 302 cattle and 57 pigs (finished on 31 March 2013) and 200 Japanese macaques.

As at the end of March 2015, we have collected samples from 302 cattle, 57 pigs, 200 Japanese macaque, 8 wild pigs and 5 horses (Fig. 6). We have identified radioactive substances and measured the radioactivity concentration in the various organs [1] and its effect on the testis [2]. Four years after the FNPP accident, we could detect ^134^Cesium (^134^Cs) and ^137^Cesium (^137^Cs) in all organs from all of the animals examined. We evaluated the effects of radiation by performing biochemical analysis of the plasma components and looking for pathological changes, and measured the radioactivity concentration in the organs from cattle obtained between August 2011 and August 2012. No significant abnormal data were found in the routine biochemical analysis. However, the cattle were suggested to be under slight stress (Urushihara *et al.*, manuscript in prep.).

The number of Japanese macaque is annually controlled at city- and village-base as a group of harmful animals. We have, therefore, collected organs from euthanized Japanese macaque. Four years after the FNPP accident, only ^134^Cs and ^137^Cs were detectable on γ-spectrometry. It is reported that pancytopenia proportional to the radio-cesium concentration in the muscle was observed in the young monkeys of Fukushima city [3]. We have been collecting monkeys from Minamisoma city and Namie town, where the radio-contamination is much higher than in Fukushima city. However, we could not observe any significant association between organ cesium radioactivity and blood cell counts (manuscript in prep.). Thyroid cancer is thought to be the only cancer that is known to have been induced by the Chernobyl accident. The number of child thyroid cancer cases began to increase 5 years after the Chernobyl accident [4]. The amount of radionuclides released by the Chernobyl accident is estimated to be 5300 PBq; the amount released by the FNPP accident is 520 PBq, one-tenth that of the Chernobyl accident. Furthermore, >80% of the radionuclides released went into the Pacific Ocean [5]. Assuming that the biological effect of radiation is related to the exposure dose, the effect of the FNPP accident on human disease, if it occurs, will not be apparent until more than 5 years after the accident. Compared with the Chernobyl accident, relatively smaller amount of the radionuclides have been deposited in the ex-evacuation zone. In the light of these facts, a long-term and vigilant study of the various animals in the ex-evacuation zone will be necessary in order to understand the late effects of radioactive cesium on the ecosystem and on humans. Detailed data are available upon request. Please contact the corresponding author (MF).

## FUNDING

This work was supported in part by the Emergency Budget for the Reconstruction of Northeastern Japan, the Ministry of Education, Culture, Sports, Science, and Technology (MEXT), Japan; the Discretionary Expense of the President of Tohoku University; the Research and Development Projects for Application in Promoting New Policy of Agriculture, Forestry and Fishers, the Ministry of Agriculture, Forestry and Fisheries (MAFF), Japan; and the Program for Promotion of Basic and Applied Research for Innovations in Bio-oriented Industry, BRAIN, Japan. Funding to pay the Open Access publication charges for this article was provided by the Grant-in-Aid from the Japan Society for the Promotion of Science (JSPS) [KAKENHI Grant No. 26253022].
